# Reduction in hsCRP levels is associated with decreased incidence of cardiovascular events in Japanese hypertensive women but not in men

**DOI:** 10.1038/s41598-020-73905-4

**Published:** 2020-10-12

**Authors:** Shokei Kim-Mitsuyama, Hirofumi Soejima, Osamu Yasuda, Koichi Node, Hideaki Jinnouchi, Eiichiro Yamamoto, Taiji Sekigami, Hisao Ogawa, Kunihiko Matsui

**Affiliations:** 1 Department of Pharmacology and Molecular Therapeutics, Graduate School of Medical Sciences, Kumamoto University, Kumamoto, 860-8556 Japan; 2 Department of Cardiovascular Medicine, Graduate School of Medical Sciences, Kumamoto University, Kumamoto, Japan; 3 Health Care Center, Kumamoto University, Kumamoto, Japan; 4 Department of Sports and Life Sciences, National Institute of Fitness and Sports in Kanoya, Kanoya, Japan; 5 Department of Cardiovascular Medicine, Saga University, Saga, Japan; 6 Diabetes Care Center, Jinnouchi Clinic, Kumamoto, Japan; 7 Division of Internal Medicine & Diabetes and Endocrine, Sekigami Clinic, Yatsushiro, Japan; 8 National Cerebral and Cardiovascular Center, Suita, Japan; 9 Department of General and Community Medicine, Kumamoto University Hospital, Kumamoto, Japan

**Keywords:** Predictive markers, Biomarkers

## Abstract

To test our hypothesis that the magnitude of reduction in hsCRP achieved by antihypertensive medications may predict the benefit for cardiovascular outcomes in hypertensive individuals, we performed subanalysis of the ATTEMPT-CVD study. The hypertensive participants enrolled in the ATTMEPT-CVD study were categorized into two groups according to whether achieved reduction in hsCRP levels at 6 months after initiation of antihypertensive medications from baseline was equal to or greater than 40% (responder group) or less than 40% (non-responder group). Baseline characteristics and blood pressure during follow-up period were similar between the groups. For women, the incidence of cardiovascular events was significantly less in responder group than non-responder group (P < 0.0221). However, for men, there was no significant difference between the groups regarding incident cardiovascular events (P = 0.2434). There was a significant interaction (P = 0.0187) between sexes for incident cardiovascular events. Our results provide the evidence suggesting that substantial reduction (40% or greater reduction) in hsCRP on antihypertensive medication predicts the benefit for cardiovascular outcomes in hypertensive women but it does not in hypertensive men. The magnitude of achieved reduction in hsCRP by antihypertensive medications seems to be a useful indicator of successful treatment in Japanese hypertensive women.

This trial was registered with ClinicalTrials.gov, number NCT01075698.

## Introduction

Chronic inflammation plays a key role in the initiation and progression of atherothrombosis, metabolic disorders including hypertension, and cardiovascular and renal diseases^1,2^. C-reactive protein (CRP) is an established inflammatory biomarker and is one of the most widely used biomarkers associated with risk of cardiovascular events^3–7^. An individual participant meta-analysis from 54 long-term prospective studies demonstrates that CRP concentrations have a continuous association with the risk of coronary heart disease, ischemic stroke, and vascular mortality^8^. Epidemiological studies show that baseline CRP levels independently predict future development of hypertension^9–11^. However, it is still unproven whether the effect of antihypertensive medications on CRP levels is associated with the benefit of these medications in cardiovascular outcomes.

We have previously performed a trial of telmisartan prevention of cardiovascular diseases (ATTEMPT-CVD) study which is a multicenter, open-label, randomized, parallel group study to compare the effect of angiotensin receptor blocker (ARB)-based antihypertensive treatment versus non-ARB-based antihypertensive treatment on the longitudinal change in various biomarkers including high-sensitivity CRP (hsCRP) and incidence of cardiovascular events in Japanese hypertensive patients with at least one cardiovascular risk^12,13^. The ATTEMPT-CVD study showed that ARB-based antihypertensive therapy caused a smaller increase in plasma brain natriuretic peptide (BNP) and a greater decrease in urinary albumin/creatinine ratio (UACR) than non-ARB-based antihypertensive therapy, despite their similar blood pressure (BP)-lowering effects^12^. However, the incidence of cardiovascular event was similar between the two therapies and the longitudinal change in hsCRP levels during 3-year follow-up period was also comparable between the two therapies^12^. Thus, the positive effect of ARB-based antihypertensive therapy on BNP or UACR is not related to the benefit in cardiovascular outcomes in hypertensive patients^12^.

In contrast to lack of evidence for the effect of antihypertensive medications on CRP levels, statin medications are well known to reduce CRP levels independently of their cholesterol-lowering effects^14–19^. In JUPITER trial, the reduction in hsCRP levels with statin therapy is an important indicator of treatment success and has prognostic implications^15,16^. Furthermore, the Canakinumab Anti-inflammatory Thrombosis Outcome Study (CANTOS) trial^20–22^, which compared the effects of canakinumab, monoclonal antibody targeting interleukin-1β, with placebo on cardiovascular events and hsCRP levels in patients with previous myocardial infarction and high hsCRP levels, showed that the magnitude of on-canakinumab reduction in hsCRP levels is associated with the benefit for subsequent cardiovascular outcomes. These findings obtained by the JUPITER^15,16^ and CANTOS^21,22^ trials encouraged us to hypothesize that the magnitude of reduction in hsCRP after initiation of antihypertensive medications may predict the benefit for cardiovascular outcomes in hypertensive patients. In the present study, to test our hypothesis, we performed subanalysis of the participants enrolled in ATTEMPT-CVD trial according to the magnitude of reduction in hsCRP after initiation of antihypertensive medications.

## Results

### Time course of serum hsCRP concentration in responder group and non-responder group

**Figure 1:**
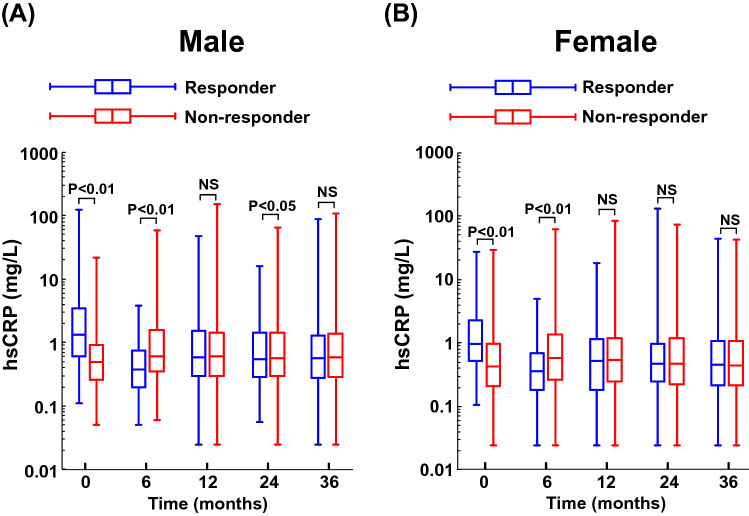
Serum hsCRP concentrations at baseline and at 6, 12, 24 and 36 months after initiation of antihypertensive medications in responder group and non-responder group for male (**A**) and female (**B**). Box plots indicate 25th percentile, median, and 75th percentile and whiskers indicate the minimum and maximum. *Responder* the group with 40% or greater reduction in hsCRP at 6 months from baseline; *Non-responder* the group with less than 40% reduction in hsCRP at 6 months from baseline; *NS* not significant.

Figure 1 shows serum hsCRP levels during follow-up period in responder and non-responder groups for male or female patients. Supplementary Table S1 indicates each actual number of median and interquartile range of Fig. 1 data. As shown in Supplementary Table S1, 192 (28.5%) of 673 male patients and 123 (25.1%) of 490 female patients were responder group (namely, group with hsCRP reduction of ≥ 40%), and the proportion of responder group was comparable between male and female (P = 0.2046). Responder group had lower 6-month hsCRP levels than non-responder group for male (6-month median hsCRP:0.37 vs 0.61 mg/L; P < 0.0005) and for female (6-month median hsCRP: 0.37 vs 0.58 mg/L; P < 0.0005). Thereafter, at 12, 24, and 36 months, for both sexes, responder group showed much the same hsCRP levels as non-responder group, except for very slight difference in 24-month hsCRP levels in male. However, baseline hsCRP levels were significantly greater in responder group than in non-responder group, for male (baseline median hsCRP: 1.31 vs 0.50 mg/L; P = 0.0016) and for female (baseline median hsCRP: 0.98 vs 0.44 mg/L; P = 0.0032).

### Baseline characteristics and prescribed medications of responder group and non-responder group

Table 1 shows demographical and baseline characteristics of responder and non-responder groups for both sexes. As described above, for both sexes, baseline hsCRP levels were significantly higher in responder group than in non-responder group. However, for both sexes, there was no difference between responder and non-responder groups, regarding baseline parameters including age, BMI, blood pressure, proportion of diabetes mellitus, hyperlipidemia, current smoker, and baseline cardiovascular disease, LDL cholesterol, HDL cholesterol, hemoglobin A1c, white blood cell number, hemoglobin, etc. Furthermore, various biomarker levels at baseline including plasma BNP, UACR, eGFR, high molecular weight adiponectin, total adiponectin, and urinary 8-OHdG were similar between the groups, for either sex. As shown in Supplementary Table S2, the proportion of prescribed ARB, other antihypertensive agents, statin, oral hypoglycemic agents, insulin, aspirin, or other antithrombotic agents did not differ between responder and non-responder groups, for either male or female.

**Table 1:** Demographical and baseline characteristics of the patients divided into two groups according to whether the achieved reduction in hsCRP at 6 months from baseline was equal to or greater than 40% (responder), or less than 40% (non-responder).

	Male (n = 673)			Female (n = 490)		
	Responder (n = 192)	Non-responder (n = 481)	P value	Responder (n = 123)	Non-responder (n = 367)	P value
hsCRP (mg/L)	1.31 (0.61–3.40)	0.50 (0.26–0.91)	< 0.0001	0.98 (0.54–2.31)	0.44 (0.21–0.97)	< 0.0001
Age (years)	64.5 ± 8.9	65.0 ± 10.1	0.2738	69.0 ± 8.3	68.3 ± 8.2	0.3492
BMI (kg/m^2^)	25.2 ± 3.3	25.1 ± 3.6	0.7201	25.0 ± 4.3	25.5 ± 4.2	0.2125
Systolic BP (mmHg)	150.5 ± 14.3	150.7 ± 16.3	0.9139	150.7 ± 14.3	149.0 ± 15.7	0.3111
Diastolic BP (mmHg)	86.1 ± 10.4	85.7 ± 11.6	0.6542	82.6 ± 12.1	80.9 ± 11.8	0.0991
Heart rate (b.p.m)	73.2 ± 12.9	71.8 ± 11.0	0.2403	73.0 ± 10.0	71.8 ± 10.5	0.1570
Diabetes mellitus, n (%)	134 (69.8)	311 (64.7)	0.2038	82 (66.7)	258 (70.3)	0.4493
Hyperlipidemia, n (%)	104 (54.2)	248 (51.6)	0.5408	88 (71.5)	239 (65.1)	0.1908
Current smoker, n (%)	46 (24.0)	131 (27.2)	0.3833	14 (11.4)	63 (17.2)	0.1271
Previous cardiovascular disease, n (%)	104 (54.2)	293 (60.9)	0.1080	63 (51.2)	191 (52.0)	0.8742
Allocation to ARB therapy, n (%)	104 (54.2)	234 (48.6)	0.1961	60 (48.8)	181 (49.3)	0.9177
Total cholesterol (mg/dL)	191.9 ± 35.3	193.8 ± 38.3	0.4776	200.4 ± 34.8	198.2 ± 32.1	0.8763
LDL cholesterol (mg/dL)	109.5 ± 28.3	111.6 ± 30.2	0.4008	114.8 ± 31.6	112.6 ± 27.5	0.6998
HDL cholesterol (mg/dL)	54.8 ± 15.1	54.4 ± 13.5	0.7850	58.5 ± 12.4	58.5 ± 13.2	0.8984
Blood sugar (mg/dL)	142.5 ± 60.0	135.4 ± 57.4	0.0327	124.1 ± 43.9	132.0 ± 52.9	0.3253
Hemoglobin A1c (%)	6.5 ± 1.2	6.3 ± 1.1	0.0626	6.3 ± 1.1	6.3 ± 1.1	0.6009
Plasma BNP (pg/mL)	16.4 (8.4–31.6)	15.5 (7.5–34.5)	0.4928	19.9 (11.3–44.2)	21.6 (11.7–38.9)	0.8509
UACR (mg/g creatinine)	31.7 (11.2–106.8)	23.00 (9.8–81.8)	0.1656	30.9 (14.9–89.4)	25.4 (11.6–81.9)	0.2221
eGFR (ml/min per 1.73 m^2^)	72.1 (62.1–88.5)	71.2 (59.6–83.5)	0.1019	70.2 (60.7–84.3)	71.0 (58.6–84.7)	0.5547
HMW adiponectin (μg/mL)	2.7 ± 2.9	2.7 ± 2.7	0.2046	4.2 ± 3.1	4.7 ± 4.5	0.5431
Total adiponectin (μg/mL)	5.4 ± 3.6	5.5 ± 3.8	0.1994	7.3 ± 4.1	8.1 ± 5.5	0.3329
Urinary 8-OHdG (ng/mL)	11.2 (7.0–16.9)	10.4 (6.3–15.4)	0.1208	9.5 (5.7–14.2)	7.9 (4.5–13.0)	0.0757
Hemoglobin (g/dL)	14.6 ± 1.38	14.6 ± 1.45	0.9598	13.0 ± 1.3	13.0 ± 1.3	0.4404
White blood cell (/μL)	6149 ± 1495	6075 ± 1614	0.3179	5924 ± 1662	5881 ± 1590	0.9094
Creatinine (mg/dL)	0.86 ± 0.25	0.88 ± 0.23	0.0885	0.65 ± 0.17	0.67 ± 0.18	0.5790
Potassium (mEq/L)	4.24 ± 0.45	4.29 ± 0.51	0.3376	4.35 ± 0.51	4.32 ± 0.55	0.3246
Uric acid (mg/dL)	5.7 ± 1.4	5.7 ± 1.3	0.9089	4.8 ± 1.3	4.8 ± 1.2	0.9147

Responder indicates the group in whom reduction of hsCRP at 6 months from baseline was equal to or greater than 40%, while Non-responder indicates the group in whom reduction of hsCRP at 6 months from baseline was less than 40%.

hsCRP, plasma BNP, UACR, eGFR, and urinary 8-OHdG are expressed as median with interquartile range. Other data are mean ± s.d. for continuous values and number (%) for categorical variables.

*hsCRP* high-sensitivity C-reactive protein, *BMI* Body Mass Index, *BP* blood pressure, *ARB therapy* antihypertensive treatment with angiotensin II receptor blocker, *LDL* low-density lipoprotein, *HDL* high-density lipoprotein, *BNP* brain natriuretic peptide, *UACR* urinary albumin/creatinine ratio, *eGFR* estimated glomerular filtration rate, *HMW adiponectin* high-molecular weight adiponectin, *8-OHdG* 8-hydroxy-2′-deoxyguanosine.

P-value was calculated using unpaired t test or Mann–Whitney test for continuous variables and χ^2^ test for categorical variables.

### Incidence of composite cardiovascular and renal events in responder group and non-responder group

**Figure 2:**
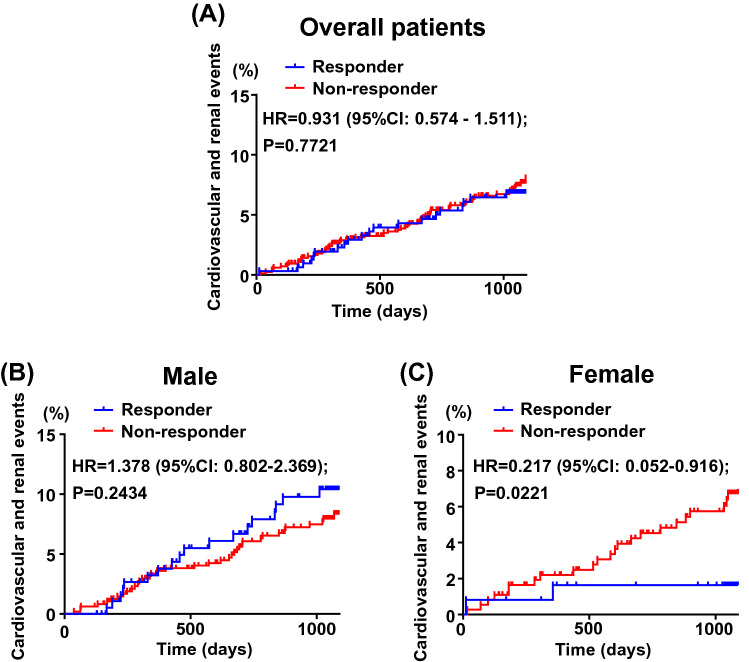
Kaplan–Meier curves for composite cardiovascular and renal events in responder group and non-responder group for overall patients (**A**), male (**B**) and female (**C**). In overall patients (**A**), there was 22 and 64 events in responder group (n = 315) and non-responder group (n = 847). In male (**B**), there was 20 and 38 events in responder group (n = 192) and non-responder group (n = 480), respectively. In female (**C**), there were 2 and 26 events in responder group (n = 123) and non-responder group (n = 367), respectively. Abbreviations used are the same as in Fig. 1.

There was no significant difference in incident cardiovascular and renal events between responder and non-responder groups of overall patients (P = 0.7721) (Fig. 2A). Subgroup analysis according to sex showed that there was no significant difference in the incidence of cardiovascular and renal events between the groups of male patients (P = 0.2434) (Fig. 2B). On the other hand, for female patients, the incidence of cardiovascular and renal events was significantly less in responder group than in non-responder group (P = 0.0221) (Fig. 2C). Furthermore, there was a significant interaction between male and female patients for the incidence of cardiovascular and renal events (P = 0.018).

### Time course of blood pressure during follow-up period

**Figure 3:**
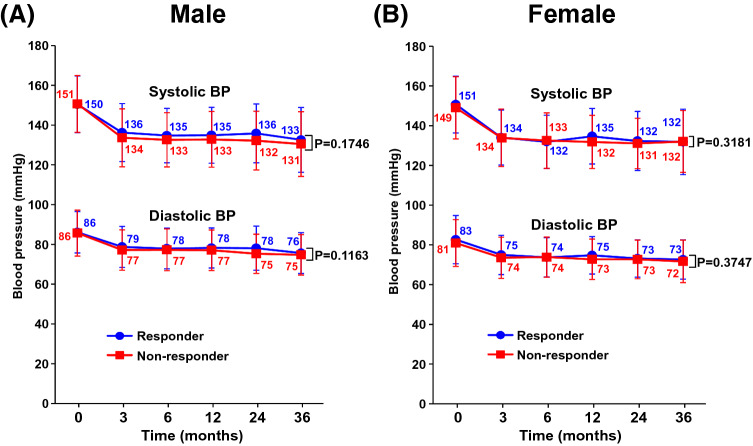
Time course of blood pressure (BP) during follow-up period in responder group and non-responder group for male (**A**) and female (**B**). Numerical values at each time point indicate the mean BP for both groups. Abbreviations used are the same as in Fig. 1.

As shown in Fig. 3, systolic and diastolic blood pressure during follow-up period was similar between responder and non-responder groups for male and female.

### Association of prognostic factors with composite cardiovascular and renal events

As shown by multivariable Cox regression analysis in Table 2, 6-month hsCRP reduction ≥ 40% (responder) was not significantly associated with composite cardiovascular and renal events in overall patients (P = 0.9041) and male patients (P = 0.1706). In contrast to male patients, 6-month hsCRP reduction ≥ 40% (responder) in female patients was significantly associated with composite cardiovascular and renal events (P = 0.0421) even after adjustment for conventional risk factors such as age, diabetes mellitus, hyperlipidemia, current smoking, and previous cardiovascular disease.

**Table 2:** Adjusted hazard ratios of prognostic factor for cardiovascular and renal events in overall, male, or female patients.

	Overall patients (n = 1162)		Male (n = 672)		Female (n = 490)	
	HR (95%CI)	P-value	HR (95%CI)	P-value	HR (95%CI)	P-value
hsCRP reduction ≥ 40%	0.971 (0.597–1.578)	0.9041	1.464 (0.849–2.524)	0.1706	0.224 (0.053–0.948)	0.0421
Sex	1.675 (1.005–2.793)	0.0478				
Age ≥ 68 years	1.513 (0.968–2.365)	0.0689	1.487 (0.877–2.522)	0.1409	1.773 (0.749–4.199)	0.1929
Diabetes mellitus	3.763 (2.139–6.621)	< 0.0001	2.892 (1.516–5.516)	0.0013	7.487 (2.139–26.20)	0.0016
Hyperlipidemia	0.904 (0.585–1.399)	0.6515	0.908 (0.540–1.525)	0.7142	0.868 (0.388–1.944)	0.7316
Smoking	0.922 (0.694–1.224)	0.5752	0.941 (0.698–1.268)	0.6896	0.571 (0.174–1.875)	0.3559
Previous CV disease	3.263 (1.988–5.355)	< 0.0001	3.147 (1.686–5.876)	0.0003	3.672 (1.627–8.289)	0.0017

hsCRP reduction ≥ 40% indicates 40% or greater reduction in hsCRP at 6 months after initiation of antihypertensive medication.

*HR*, hazard ratio, *95% CI* 95% confidence interval, *CV* cardiovascular.

### Incidence of composite cardiovascular and renal events according to quartiles of baseline serum hsCRP levels

**Figure 4:**
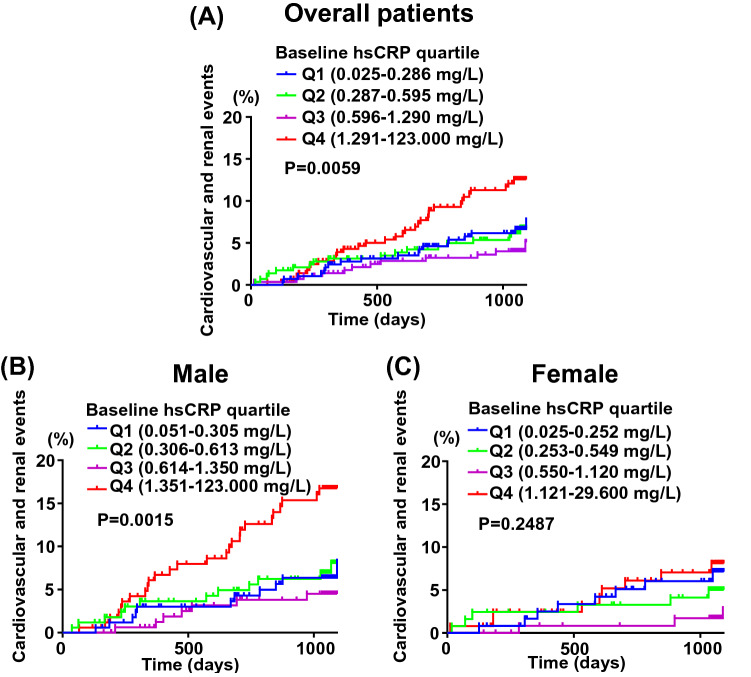
Kaplan–Meier curves for composite cardiovascular and renal events stratified by quartiles of baseline hsCRP levels for overall patients (**A**), male (**B**) and female (**C**). In overall patients (**A**), the number of occurrence of events was 21, 20, 12, and 33 in Q1 (n = 292), Q2 (n = 290), Q3 (n = 290), and Q4 (n = 290), respectively. In male (**B**), the number of occurrence of events was 12, 13, 7, and 26 in Q1 (n = 168), Q2 (n = 168), Q3 (n = 167), and Q4 (n = 169), respectively. In female (**C**), the number of occurrence of events was 9, 7, 3, and 9 in Q1 (n = 122), Q2 (n = 123), Q3 (n = 121), and Q4 (n = 124), respectively.

As shown in Fig. 4A, a significant difference in the incidence of cardiovascular and renal events was found among overall patients with quartiles of baseline hsCRP levels (P = 0.0059). Subgroup analysis according to sex showed that in the case of male, there was a significant difference in the incidence of cardiovascular and renal events among quartiles of baseline hsCRP (P = 0.0015) and patients with the highest baseline hsCRP (Q4) had more cardiovascular events (Fig. 4B). On the other hand, there was no significant difference in incident cardiovascular events among female patients with quartile of baseline hsCRP levels (P = 0.2487) (Fig. 4C).

## Discussion

Our present post-hoc analysis showed no significant association of hsCRP reduction with cardiovascular and renal events in hypertensive patients. However, subgroup analysis according to sex showed that substantial reduction (40% or greater reduction) in hsCRP achieved by antihypertensive medications is associated with the significant reduction of cardiovascular events in hypertensive women, whereas it was not true in hypertensive men. Hence, it is possible that the assessment of magnitude of hsCRP reduction after initiation of antihypertensive medication seems to be useful for predicting successful treatment in hypertensive women.

To the best of our knowledge, the relationship between the magnitude of reduction in hsCRP on antihypertensive medication and subsequent cardiovascular outcomes in hypertensive individuals has not been reported. In contrast to the uncertainty in the effect of antihypertensive medications on hsCRP levels^10,23–25^, statin therapy has been shown to lower circulating hsCRP levels in various populations^3,14,15,17,19,26,27^. In the JUPITER trial^15,16^, which enrolled apparently healthy individuals with low LDL cholesterol but with an elevated hsCRP, statin (rosuvastatin 20 mg) reduced hsCRP levels by 37%, and significantly reduced the incidence of major cardiovascular events. Intriguingly, the magnitude of achieved reduction in hsCRP on rosuvastatin therapy predicts the benefit in future cardiovascular outcomes independently of LDL cholesterol-lowering effect^16^. By contrast, in ASCOT trial^28^ of participants with no previous coronary heart disease but with hypertension and 3 or more previous classical cardiovascular risk factors, statin (atorvastatin 10 mg) therapy reduced hsCRP levels by only 27% (by a lesser extent than 37% reduction in JUPITER trial^16^), and 27% on-treatment reduction in hsCRP levels does not predict subsequent cardiovascular outcomes, thereby suggesting that greater-than-27% on-statin reduction in hsCRP might be necessary to cause the benefit in cardiovascular outcomes in these participants. Furthermore, recently, to test the inflammatory hypothesis of atherothrombosis, CANTOS trial^21,22^ have investigated the effect of canakinumab, a monoclonal antibody targeting interleukin-1β, on major cardiovascular events in patients with a previous history of myocardial infarction and with high hsCRP levels compared with placebo and this trial have also addressed the relationship between the magnitude of on-treatment reduction in hsCRP and reduction of cardiovascular events. CANTOS trial showed that canakinumab therapy at doses of either 150 or 300 mg resulted in a 39% achieved reduction in hsCRP and a significant reduction in major adverse cardiovascular events compared with placebo, whereas canakinumab at a lower dose (50 mg) resulted in only 26% reduction in hsCRP and non-significant reduction of cardiovascular events, supporting that intensive reduction of hsCRP (namely, intensive amelioration of inflammation) is necessary for the significant reduction of cardiovascular events. Furthermore, this trial shows that the magnitude of achieved hsCRP reduction by canakinumab therapy relates to the magnitude of clinical benefit for incident cardiovascular events^22^. Based on these findings on the magnitude of achieved hsCRP reduction on statin therapy^15^ or on canakinumab therapy^21,22^, in the present study, we hypothesized that 40% or greater reduction in hsCRP after initiation of antihypertensive medication may predict the benefit for cardiovascular outcomes in hypertensive individuals. To test this hypothesis, we categorized hypertensive participants enrolled in the ATTEMPT-CVD study into two groups according to on-treatment reduction in hsCRP of ≥ 40% (responder) or < 40% (non-responder). Importantly, the prespecified secondary endpoint of the ATTEMPT-CVD study has included the longitudinal change in hsCRP during follow-up period, which allowed us to perform the present post-hoc analysis. Notably, responder group was characterized by higher hsCRP levels at baseline before initiation of antihypertensive medication compared with non-responder group (P < 0.01 for either men or women). Despite higher hsCRP levels at baseline, female responder group experienced significantly less cardiovascular events than female non-responder group. Moreover, 40% or greater on-treatment reduction in hsCRP (responder) was a significant independent prognostic factor for cardiovascular events in hypertensive women even after adjustment for conventional risk factors. Thus, our work provides the evidence suggesting that the substantial reduction (40% or greater reduction) in hsCRP achieved by antihypertensive medications may be a useful predictor of benefit in subsequent cardiovascular outcomes in hypertensive women. However, at present, the definition of hsCRP responder has not been established in hypertensive population. Different cutoff values of hsCRP such as 50% reduction in hsCRP or on-treatment hsCRP levels of < 2.0 mg/L or < 1.8 mg/L have been also used in other populations^16,20,22^. Furthermore, being consistent with previous reports^29–32^, hsCRP levels are much lower in Japanese population than in Western population. Therefore, further study is needed to elucidate which cutoff value is most suitable for definition of hsCRP responder.

It is a key issue to address the potential mechanism underlying the significant reduction of cardiovascular events in hypertensive female responder group. Compelling evidence indicates a critical role of inflammation in all steps of the atherosclerotic process^1,6^. Taken together with the fact that CRP is a highly reliable marker of systemic inflammation^5,14,19^, our present findings suggest that less incidence of cardiovascular events in female responder group might be at least partially mediated by the significant amelioration of inflammation. Alternatively, it is possible that less incidence of cardiovascular events in hypertensive female responder group might be partially attributed to the improvement of life style caused by Hawthorne effect^33^. Moreover, the difference in estrogen levels among women might partially affect the incidence of cardiovascular events because estrogen significantly affects CRP levels^34^. Further study is needed to elucidate the precise mechanism of our present findings.

Prospective cohort study shows that hsCRP levels at baseline predict the development of new-onset hypertension^9,11^. However, it has been uncertain whether antihypertensive medications significantly alter circulating hsCRP levels in hypertensive individuals and whether their effects on hsCRP levels differ among different classes of antihypertensive medications. In the present study, we found that the magnitude of 6-month reduction in hsCRP after initiation of antihypertensive medication considerably varies among hypertensive individuals, and we identified a subset of hypertensive men and women who displayed 40% or greater reduction in hsCRP after initiation of antihypertensive medications. It is possible that such considerable variation in on-treatment reduction in hsCRP observed in this study might be attributed to the difference in baseline characteristics, the difference in blood pressure lowering, or the use of different classes of antihypertensive agents among individuals. Importantly, except for baseline CRP levels, baseline characteristics were well balanced between responder and non-responder groups, thereby validating the findings of our present subanalysis. Blood pressure not only at baseline but also during follow-up period was similar between responder and non-responder groups. There was no significant difference between the groups regarding the characteristics of prescribed antihypertensive agents. Furthermore, the proportion of prescribed agents potentially affecting hsCRP levels, such as statin, aspirin, antidiabetic agents, etc. did not differ between the groups. Hence, it is unlikely that considerable variation in on-treatment percentage reduction in hsCRP levels among hypertensive individuals might be due to the difference in blood pressure levels or the difference in characteristics of prescribed antihypertensive medications or other medications between the groups. On the other hand, it is possible that considerable variation in hsCRP reduction observed in this study might be partially attributed to the difference in life style or environmental factor among individuals.

It has been shown that there is significant heterogeneity between sexes regarding the association of hsCRP with cardiovascular events^19,35^, although the detail on sex difference remains to be clarified. Previous analysis comprising individual participant data from 52 prospective cohort studies show that association of hsCRP with coronary heart disease may be less in women than in men, and the net improvement in risk reclassification with the use of hsCRP was larger for men compared with women^19,35^. Our present analysis by quartiles of baseline hsCRP demonstrated the significant association of high hsCRP at baseline with the increased cardiovascular events in hypertensive men, and this finding on men is in good agreement with previous findings on the association of baseline CRP levels with cardiovascular events in other various populations^5,7,8,14,18,29,32^. On the other hand, in the case of women, we found no significant association between baseline hsCRP levels and cardiovascular outcomes. Interestingly, there is no significant association between baseline hsCRP and incident ischemic stroke for the women in both Japanese^32^ and Western^36^ populations. Taken together with previous findings^19,32,35,36^, our present results suggest less association of baseline hsCRP with cardiovascular outcomes in women than in men, although it cannot be excluded that no association of baseline hsCRP with cardiovascular outcome in women might be attributed to the small sample size.

### Study limitation

There are several study limitations in this study. First, this study was a pos-hoc analysis of ATTEMPT-CVD study, although longitudinal change in hsCRP was a prespecified secondary endpoint. Second, the present study did not permit us to elucidate the underlying mechanism by which the magnitude of hsCRP reduction after initiation of antihypertensive medications considerably varied among hypertensive patients, despite their similar blood pressure reduction. Third, it cannot be completely excluded that blood pressure-independent factors, such as environmental, behavioral, estrogen levels, or genetic factor, might be partially responsible for such variation in hsCRP reduction among individuals, because these factors are known to modify hsCRP levels^10,34,37^. Fourth, it cannot be also completely ruled out that CRP might have a direct role in our present study, although there is still controversy on whether CRP per se has vascular or prothrombotic effects or is simply a marker of inflammation^10^. Fifth, it cannot be completely excluded that there might be a type I statistical error in this post-hoc analysis because of multiple analyses. However, in this study, there was a significant interaction between male and female patients for the incidence of cardiovascular and renal events, and hsCRP reduction ≥ 40% (responder) in female patients was significantly associated with composite cardiovascular and renal events even after adjustment for conventional risk factors. We believe that these findings support our interpretation. Finally, the cohort of the ATTEMPT-CVD study was limited to Japanese hypertensive patients. Given great variability in hsCRP levels among ethnicities and lower hsCRP levels in Japanese than Western populations^10,29,31,37^, it is unclear whether the present findings apply to Western hypertensive patients, although the trial on only Japanese patients had the advantage of fewer confounding factors compared with a world-wide trial.

In conclusion, on-treatment hsCRP reduction was not significantly associated with the incidence of cardiovascular events in Japanese hypertensive patients. However, subanalysis provided the evidence suggesting that substantial reduction in hsCRP after initiation of antihypertensive medication was associated with the significant reduction of cardiovascular events in hypertensive women but it was not true in hypertensive men. Prospective randomized larger trial is needed to validate our observations, since our present findings are hypothesis-generating.

## Methods

### Study design and participants of ATTEMPT-CVD study

This study is a post-hoc subanalysis of a trial of telmisartan prevention of cardiovascular disease (ATTEMPT-CVD). Study design^13^ and the main results^12^ of ATTEMPT-CVD study have been described in our previous reports. Briefly, the ATTEMPT-CVD study was a multicenter, prospective, randomized, open-label, blinded endpoint, active-controlled trial to compare the effects of an angiotensin receptor blocker (ARB)-based antihypertensive therapy and those of non-ARB antihypertensive therapy on longitudinal changes in various biomarker and the incidence of composite cardiovascular and renal events in hypertensive outpatients with at least one cardiovascular risk. Between July 2009 and April 2011, 1228 patients were enrolled from 168 institutions throughout Japan. Each patient was followed up for three years.

Patients were eligible if they were 40 to 79-year-old hypertensive outpatients with at least one cardiovascular risk (type 2 diabetes, or cardiac factors, cerebral factors, peripheral arterial, or renal factors). The exclusion criteria consisted of type 1 diabetes, severe renal disorder (serum creatinine > 2.0 mg/dL), heart failure (NYHA Class III or IV), myocardial infarction, percutaneous revascularization and bypass grafting of coronary artery/lower extremity vessels, cerebral infarction, cerebral hemorrhage, subarachnoid hemorrhage, or transient cerebral ischemic attack within 6 months before enrollment. The following patients were also excluded: patients with malignant hypertension, secondary hypertension, pregnant women, clinically problematic allergic disease or past history of hypersensitivity to the drugs used, past history of significant adverse drug reactions, extremely poor biliary secretion or serious hepatic disorder, patients who require treatment for a malignant tumor, and other patients who are judged by the physician to be unsuitable for the study. Full inclusion and exclusion criteria are provided in our previous protocol paper^13^. The study protocol was approved by Independent Ethics Committee of Kumamoto University and complied with the Declaration of Helsinki. The institutional review board of each participating hospital approved this trial, and written informed consent was obtained from each patient.

The eligible patients were randomly assigned in a 1:1 ratio by a computer-generated stratified randomization sequence to be allocated to either the ARB (telmisartan) treatment group or the non-ARB treatment group, as described^12,13^. Follow-up period was 3 years. At study registration and at 6, 12, 24 and 36-month after the initiation of antihypertensive therapy, urinary albumin/creatinine ratio (UACR), plasma brain natriuretic peptide (BNP), serum high-sensitive C-reactivity protein (hsCRP), urinary 8-hydroxy-deoxy-guanosine (8-OHdG), serum adiponectin, serum high molecular weight adiponectin, estimated glomerular filtration rate (eGFR) were estimated to compare between ARB-based antihypertensive treatment and non-ARB antihypertensive treatment.

### Endpoints

The primary endpoints of the ATTEMPT-CVD study was to compare between ARB-based and non-ARB-based treatments regarding longitudinal changes in UACR and in plasma BNP concentrations from baseline^12,13^. The prespecified secondary endpoints were to compare between the groups regarding the changes in serum hsCRP, urinary 8-OHdG, serum total adiponectin, serum high molecular weight adiponectin, and eGFR, and the incidence of composite cardiovascular and renal events (time until occurrence of events)^12,13^. Cardiovascular and renal events consisted of cerebral events (cerebral infarction, cerebral hemorrhage, subarachnoid hemorrhage, unknown type of stroke, transient ischemic attack), cardiac events (sudden death, myocardial infarction, angina pectoris, asymptomatic myocardial ischemia, heart failure), aortic/peripheral arterial events (aortic aneurysm, aortic dissection, arteriosclerotic disease), newly occurred or aggravated diabetic complications (diabetic nephropathy, diabetic retinopathy, diabetic neuropathy), aggravation of renal function (doubling of serum creatinine, initiation of dialysis, renal transplantation).

### Subanalysis

In the main subanalysis, for each sex, the participants were classified into two groups according to whether achieved reduction in hsCRP levels at 6 months after initiation of antihypertensive medications from baseline was equal to or greater than 40% (defined as “responder group”) or less than 40% (defined as “non-responder group”).

In another subanalysis, baseline serum hsCRP concentration was categorized by quartiles (Q1, Q2, Q3, and Q4) for each sex.

### Measurement of serum hsCRP

All biomarkers and laboratory analyses were performed in SRL, Inc. (Tokyo, Japan). Serum hsCRP concentrations were measured with high-sensitivity nephelometry assay.

### Statistical analyses

The rationale of sample size and power calculation for the original study has been described^12,13^. All analyses were performed in compliance with intention-to-treat principle. Two-way repeated measures analysis of variance was used to compare between responder group and non-responder group for time course of serum hsCRP concentrations and for time course of systolic or diastolic blood pressure during the follow-up period. The unpaired t-test adjusted by Holm’s method was used for intergroup comparison to avoid multiplicity at multiple time points. As for cardiovascular and renal events, time to first event curves were estimated by the Kaplan–Meier method, and the log-rank test was used to analyze the differences between responder and non-responder groups regarding the incidence of cardiovascular events. Kaplan–Meier method and the log-rank test were also used to analyze the difference among patients with quartiles of baseline serum hsCRP. Using a stratified Cox proportional hazard model, the hazard ratio (HR) and its 95% confidence interval (CI) were calculated for each group. To determine the relationship between prognostic factors and the incidence of cardiovascular events, a multivariable Cox proportional hazards analysis was performed for different population models (overall, male, and female participants) after adjusting for the following covariates: sex, age, current smoking, baseline diabetes, baseline hyperlipidemia, and baseline cardiovascular disease. To compare the difference in baseline parameters between responder and non-responder groups for either sex, P-value was calculated using unpaired t test or Mann–Whitney test for continuous variables and using χ^2^ tests for categorical variables. Windows SAS Version 9.2 and subsequent versions were used as the statistical analysis software. P-values of less than 0.05 were considered statistically significant.

## Supplementary information

Supplementary Information.

## Data Availability

The datasets generated during and/or analysed during the current study are available from the corresponding author on reasonable request.

## References

[1] Libby P, Ridker PM, Hansson GK, Leducq Transatlantic Network on Atherothrombosis (2009). Inflammation in atherosclerosis: From pathophysiology to practice. J. Am. Coll. Cardiol..

[2] Hansson GK (2005). Inflammation, atherosclerosis, and coronary artery disease. N. Engl. J. Med..

[3] Koenig W (2013). High-sensitivity C-reactive protein and atherosclerotic disease: From improved risk prediction to risk-guided therapy. Int. J. Cardiol..

[4] Liuzzo G (1994). The prognostic value of C-reactive protein and serum amyloid a protein in severe unstable angina. N. Engl. J. Med..

[5] Myers GL (2004). CDC/AHA Workshop on markers of inflammation and cardiovascular disease: Application to clinical and public health practice: Report from the laboratory science discussion group. Circulation.

[6] Ridker PM, Cushman M, Stampfer MJ, Tracy RP, Hennekens CH (1997). Inflammation, aspirin, and the risk of cardiovascular disease in apparently healthy men. N. Engl. J. Med..

[7] Sabatine MS (2007). Prognostic significance of the Centers for Disease Control/American Heart Association high-sensitivity C-reactive protein cut points for cardiovascular and other outcomes in patients with stable coronary artery disease. Circulation.

[8] Emerging Risk Factors Collaborations (2010). C-reactive protein concentration and risk of coronary heart disease, stroke, and mortality: An individual participant meta-analysis. Lancet.

[9] Dauphinot V (2009). C-reactive protein implications in new-onset hypertension in a healthy population initially aged 65 years: The proof study. J. Hypertens..

[10] Hage FG (2014). C-reactive protein and hypertension. J. Hum. Hypertens..

[11] Sesso HD (2003). C-reactive protein and the risk of developing hypertension. JAMA.

[12] Ogawa H (2016). A trial of telmisartan prevention of cardiovascular diseases (ATTEMPT-CVD): Biomarker study. Eur. J. Prev. Cardiol..

[13] Soejima H (2014). The changes of biomarkers by telmisartan and their significance in cardiovascular outcomes: Design of a trial of telmisartan prevention of cardiovascular diseases (ATTEMPT-CVD). J. Clin. Trials.

[14] Ridker PM (2016). A test in context: High-sensitivity C-reactive protein. J. Am. Coll. Cardiol..

[15] Ridker PM (2008). Rosuvastatin to prevent vascular events in men and women with elevated C-reactive protein. N. Engl. J. Med..

[16] Ridker PM (2009). Reduction in C-reactive protein and LDL cholesterol and cardiovascular event rates after initiation of rosuvastatin: A prospective study of the JUPITER trial. Lancet.

[17] Ridker PM (2001). Measurement of C-reactive protein for the targeting of statin therapy in the primary prevention of acute coronary events. N. Engl. J. Med..

[18] Ridker PM, Rifai N, Rose L, Buring JE, Cook NR (2002). Comparison of C-reactive protein and low-density lipoprotein cholesterol levels in the prediction of first cardiovascular events. N. Engl. J. Med..

[19] Yousuf O (2013). High-sensitivity C-reactive protein and cardiovascular disease: A resolute belief or an elusive link?. J. Am. Coll. Cardiol..

[20] Everett BM (2019). Anti-inflammatory therapy with canakinumab for the prevention of hospitalization for heart failure. Circulation.

[21] Ridker PM (2017). Antiinflammatory therapy with canakinumab for atherosclerotic disease. N. Engl. J. Med..

[22] Ridker PM (2018). Relationship of C-reactive protein reduction to cardiovascular event reduction following treatment with canakinumab: A secondary analysis from the CANTOS randomised controlled trial. Lancet.

[23] Fulop T (2009). C-reactive protein among community-dwelling hypertensives on single-agent antihypertensive treatment. J. Am. Soc. Hypertens..

[24] Palmas W (2007). Antihypertensive medications and C-reactive protein in the multi-ethnic study of atherosclerosis. Am. J. Hypertens..

[25] Ridker PM, Danielson E, Rifai N, Glynn RJ, Val MI (2006). Valsartan, blood pressure reduction, and C-reactive protein: Primary report of the Val-MARC trial. Hypertension.

[26] Braunwald E (2012). Creating controversy where none exists: The important role of C-reactive protein in the care, AFCAPS/TexCAPS, prove it, reversal, A to Z, JUPITER, heart protection, and Ascot trials. Eur. Heart J..

[27] Heart Protection Study Collaborative Group (2011). C-reactive protein concentration and the vascular benefits of statin therapy: An analysis of 20,536 patients in the heart protection study. Lancet.

[28] Sever PS (2012). Evaluation of C-reactive protein prior to and on-treatment as a predictor of benefit from atorvastatin: Observations from the Anglo-Scandinavian cardiac outcomes trial. Eur. Heart J..

[29] Arima H (2008). High-sensitivity C-reactive protein and coronary heart disease in a general population of Japanese: The Hisayama study. Arterioscler. Thromb. Vasc. Biol..

[30] Ishikawa J (2007). Low-grade inflammation is a risk factor for clinical stroke events in addition to silent cerebral infarcts in Japanese older hypertensives: The Jichi Medical School ABPM study, wave 1. Stroke.

[31] Matsushita K (2007). High-sensitivity C-reactive protein is quite low in Japanese men at high coronary risk. Circ. J..

[32] Wakugawa Y (2006). C-reactive protein and risk of first-ever ischemic and hemorrhagic stroke in a general Japanese population: The Hisayama study. Stroke.

[33] Bellomo R, Egi M (2005). Glycemic control in the intensive care unit: Why we should wait for NICE-SUGAR. Mayo Clin. Proc..

[34] Walsh BW (2000). The effects of hormone replacement therapy and raloxifene on C-reactive protein and homocysteine in healthy postmenopausal women: A randomized, controlled trial. J. Clin. Endocrinol. Metab..

[35] Emerging Risk Factors Collaboration (2012). C-reactive protein, fibrinogen, and cardiovascular disease prediction. N. Engl. J. Med..

[36] Cao JJ (2003). C-reactive protein, carotid intima-media thickness, and incidence of ischemic stroke in the elderly: The cardiovascular health study. Circulation.

[37] Hage FG, Szalai AJ (2007). C-reactive protein gene polymorphisms, C-reactive protein blood levels, and cardiovascular disease risk. J. Am. Coll. Cardiol..

