# Supplementary material for: Reduction in hsCRP levels is associated with decreased incidence of cardiovascular events in Japanese hypertensive women but not in men
Source: Sci Rep. 2020 Oct 12;10:17040. doi: 10.1038/s41598-020-73905-4 (PMC7550334; doi:10.1038/s41598-020-73905-4)
Supplement: Supplementary file 1 — Supplementary Information. [file 41598_2020_73905_MOESM1_ESM.pdf]

Supplementary Information

Reduction in hsCRP levels is associated with decreased incidence of cardiovascular events in Japanese hypertensive women but not in men

Shokei Kim-Mitsuyama*[1], Hirofumi Soejima[2,3], Osamu Yasuda[4], Koichi Node[5], Hideaki Jinnouchi[6], Eiichiro Yamamoto[2], Taiji Sekigami[7], Hisao Ogawa[8], Kunihiko Matsui[9]

[1]Department of Pharmacology and Molecular Therapeutics, Graduate School of Medical Sciences, Kumamoto University, Kumamoto, Japan; [2]Department of Cardiovascular Medicine, Graduate School of Medical Sciences, Kumamoto University, Kumamoto, Japan; [3]Health Care Center, Kumamoto University, Kumamoto, Japan; [4]Department of Sports and Life Sciences, National Institute of Fitness and Sports in Kanoya, Kanoya, Japan; [5]Department of Cardiovascular Medicine, Saga University, Saga, Japan; [6]Diabetes Care Center, Jinnouchi Clinic, Kumamoto, Japan; [7]Division of Internal Medicine & Diabetes and Endocrine, Sekigami Clinic, Yatsushiro, Japan; [8]National Cerebral and Cardiovascular Center, Suita, Japan; [9]Department of General and Community Medicine, Kumamoto University Hospital, Kumamoto, Japan

Supplementary Information

Supplementary Table 1. Time course of during follow-up period in male or female patients divided into two groups according to whether the reduction in hsCRP at 6 months from baseline was 40% or greater (Responder), or less than 40% (Non-responder)

Time (months)	Male (n=673)			Female (n=490)		
	Responder (n=192)	Non-responder (n=481)	P value	Responder (n=123)	Non-responder (n=367)	P value
0 (Baseline)	1.31 (0.61-3.40)	0.50 (0.26-0.91)	0.0016	0.98 (0.54-2.31)	0.44 (0.21-0.97)	0.0032
6	0.37 (0.20-0.75)	0.61 (0.36-1.60)	<0.0005	0.37 (0.18-0.70)	0.58 (0.27-1.37)	<0.0005
12	0.59 (0.30-1.53)	0.61 (0.30-1.42)	0.674	0.52 (0.19-1.14)	0.55 (0.25-1.19)	0.2154
24	0.55 (0.28-1.40)	0.56 (0.29-1.43)	0.0264	0.47 (0.25-0.97)	0.49 (0.23-1.19)	0.761
36	0.57 (0.28-1.28)	0.58 (0.29-1.36)	1	0.46 (0.22-1.10)	0.45 (0.22-1.09)	0.6326

Responder, the group with 40% or greater reduction in hsCRP at 6 months from baseline; Non-responder, the group with less-than 40% reduction in hsCRP at 6 months from baseline. Serum hsCRP levels are expressed as median (interquartile range).

Supplementary Table 2. Medication characteristics at baseline in the group with hsCRP reduction ≥40% (Responder) and that with hsCRP reduction <40% (Non-responder)

	Male (n=673)			Female (n=490)		
	Responder (n=192)	Non-responder (n=481)	P value	Responder (n=123)	Non-responder (n=367)	P value
Antihypertensive agents						
ARB, n (%)	105 (54.7)	235 (48.9)	0.1719	61 (49.6)	181 (49.3)	0.9579
ACEI, n (%)	13 (6.8)	42 (8.7)	0.4017	6 (4.9)	35 (9.5)	0.1063
CCB, n (%)	120 (62.5)	292 (60.7)	0.6664	74 (60.2)	250 (68.1)	0.1066
β blockers, n (%)	26 (13.5)	83 (17.3)	0.2376	11 (8.9)	53 (14.4)	0.1173
Diuretics, n (%)	17 (8.9)	58 (12.1)	0.2330	16 (13.0)	55 (15.0)	0.5896
Others, n (%)	5 (2.6)	17 (3.5)	0.5400	4 (3.3)	19 (5.2)	0.3823
Lipid-lowering agents						
Statin, n (%)	66 (34.4)	162 (33.7)	0.8634	69 (56.1)	198 (54.0)	0.6791
Other agents, n (%)	7 (8.9)	40 (8.3)	0.8209	14 (11.4)	21 (5.7)	0.0349
Oral hypoglycemic agents, n (%)	105 (54.7)	243 (50.5)	0.3286	55 (44.7)	184 (50.1)	0.2979
Insulin therapy, n (%)	11 (5.7)	25 (5.2)	0.7819	5 (4.1)	30 (8.2)	0.1256
Anti-thrombotic agents						
Aspirin, n (%)	43 (22.4)	113 (23.5)	0.7607	30 (24.4)	80 (21.8)	0.5510
Other agents, n (%)	29 (15.1)	74 (15.4)	0.9273	12 (9.8)	40 (10.9)	0.7217

Abbreviations: ARB, angiotensin receptor blocker; ACEI, angiotensin-converting enzyme inhibitor; CCB, calcium channel blocker. P-value was calculated using χ^2 test.

Supplementary Information

A Trial of Telmisartan Prevention of Cardiovascular Diseases (ATTEMPT-CVD) Investigators

Steering Committee: Hisao Ogawa, Shokei Kim-Mitsuyama, Koichi Node, Hideaki Jinnouchi, and Taiji Sekigami

Committee on Validation of Data and Events: Sohichi Uekihara, Yoichiro Hashimoto, and Shuichi Oshima

Safety Monitoring Board: Kazuteru Fujimoto, Toshiro Yonehara, and, Teruyuki Hirano

Study Statistician: Kunihiko Matsui

Independent Data Management Center: Kaori Harada, Nobue Ueno, Yoshiko Terasaki, Mami Okamoto, Kanae Kanamori, Naoko Oka, Nami Matsuo, and, Kaori Sakaguchi

Investigators: Hirofumi Soejima, Osamu Yasuda, Megumi Yamamuro, Eiichiro Yamamoto, Yoichi Hanaoka, Hirofumi Kan, Tomio Jinnouchi, Syuichi Matsuo, Yutaka Wakasa, Naoto Yokota, Kazuo Matsunaga, Takatoshi Otonari, Toshihiko Sakanashi, Masanori Nishiyama, Hiroo Miyagi, Satoru Suzuki, Shukuko Ebihara, Masayasu Yamamoto, Ryo Fukami, Yoshimi Ohshima, Masahisa Ori, Junji Shibata, Daisuke Fujimatsu, Kouichi Mizobe, Michio Mizobe, Masato Nishimura, Shigeki Gondo, Juniti Miyata, Natsuki Nakamura, Koichi Kikuta, Yasuhiro Sakamoto, Shirou Mimori, Takashi Ono, Kunihiro Ohmori, Hirofumi Naitoh, Yasushi Suzuki, Shinichi Hirota, Kohji Honjo, Yoji Yamasaki, Kazushi Serikawa, Hideo Ikeda, Kazuo Ibaraki, Shigemasa Hashimoto, Sueo Momosaki, Satoshi Abe, Motoki Yoshinari, Kouji Sasaki, Masamitsu Toihata, Eiko Kobayashi, Takuo Ogawa, Shu Konno, Yuji Tanaka, Naoki

Yamamoto, Kazuya Shigenobu, Hiromi Fujii, Kazunori Murakami, Osamu Doi, Mari Kashiwabara, Kiyoharu Itoh, Fumihiro Oishi, Azusa Ikegami, Takao Mishima, Jouji Yamauchi, Shoichi Kitano, Hiroshi Haruguchi, Tomohiro Katsuya, Shunsuke Take, Sadao Nakajima, Haruaki Uchiyama, Yoshihiro Yamada, Munetaka Yamaguchi, Eiichiro Honda, Shinichi Uemura, Yasuhiro Morikami, Teruhiko Ito, Yoko Oe, Takashi Fukunaga, Yoshikuni Haraguchi, Masahiko Tsuji, Shigeru Kiyama, Kazuo Kuroki, Shigeya Tanaka, Kenichi Ashihara, Kenzo Motoki, Kenya Kusunose, Junko Hotchi, Hisashi Shimono, Kenichiro Iimori, Hisashi Watanabe, Fujio Yoshida, Kazuo Machii, Hiroshi Yamamoto, Kazuhisa Kodama, Atsushi Hiwatashi, Sumio Komatsu, Nakayasu Wake, Takashi Ise, Senshu Hifumi, Jun Ohbayashi, Shojiro Naomi, Yutaka Horio, Ken Iwai, Toshiro Matsunaga, Hiroshi Sakamoto, Yoshinobu Morikawa, Eisaku Harada, Yoshinobu Murasato, Masataka Horiuchi, Naomi Sugahara, Soichi Honda, Shingo Shoji, Masanori Shida, Toshinori Utsunomiya, Takao Baba, Moronari Matsunaga, Kazuyoshi Noda, Yoichi Setoguchi, Kohei Yamaguchi, Masayoshi Koga, Masashi Sakai, Yoshihisa Tamazaki, Genjiro Sato, Takanobu Nakajima, Masayuki Otani, Atsuyuki Wada, Jun Yamagami, Shinji Tamaki, Mitsuo Morita, Masako Waki, Tsukasa Katsuki, Kenji Sadamatsu, Yasuaki Koga, Shinsuke Tsuji, Yoshito Inobe, Kenji Misumi, Hiroto Okubo, Hirofumi Matsuda, Koichiro Kataoka, Kyoji Takaoka, Osamu Hashiguchi, Tomohiro Sawada, Tomonori Kanazawa, Kazuhiko Matsuo, Kunio Idegami, Eiichiro Tanaka, Yoshio Horita, Satoru Horita, Akira Maki, Koji Sasaki, Tomoki Nakamura, Masaru Doi, Katsunori Kawamitsu, Hirofumi Maeda, Takaoki Otsuka, Hiroyuki Tanaka, Takamasa Iwasawa, Kensei Hayashida, Hiroyuki Tanaka, Katuya Oosima, Yasuji Doi, Yasuhiro Nishiyama, Masashi Ikushima, Toru Kinugawa, Kaname Akioka, Yoko Anbe, Hiroaki Kono, Yasuhiro Sasaki, Mario Yamaki, Takashi Honda, Atsushi Sato, Takahiro

Hayashi, Yoshihiro Kimura, Aya Shiraki, Shunichi Sugimoto, Yasuharu Kodama, Hideki Wakamatsu, Shiho Enomoto, Yoshiyasu Yamatsu, Sadaaki Okamoto, Hiroaki Hosokawa, Kouji Maeno, Yohsuke Hanaoka, Hisanori Kanazawa, Tadasuke Chitose, Taishi Nakamura, Junichi Matsubara, Shinsuke Hanatani, Kenichi Tsujita, Satoshi Araki, Hiroaki Kusaka, Kenshi Yamanaga, Kensuke Toyama
